# 

**Published:** 1858-08

**Authors:** 


Vol. 1, AUGUST, 1858. No. 2.

### THE DENTAL REPORTER:

AN INDEPENDENT JOURNAL,

DEVOTED TO

### Dental Progress, and Improvements

IN THE

MANUFACTURE AND USE OF

INSTRUMENTS AND MATERIALS.

EDITED BY JNO. T. TOLAND.

Published Quarterly by

**JNO. T. TOLAND,**

MANUFACTURER OF

DENTAL INSTRUMENTS AND WHOLESALE DEALER IN DENTISTS' MATERIALS.

**38 West Fourth Street, Cincinnati.**

*PRICE* **25** *CENTS A YEAR IN* **ADVANCE,** *SINGLE COPIES* **10** *CENTS.*

WRIGHTSON & Co., Printers, 167 Walnut St., Cin.

